# Childhood Visual Impairment and other Challenges in the Middle East and African Region

**DOI:** 10.4103/0974-9233.71583

**Published:** 2010

**Authors:** Deepak P. Edward

**Affiliations:** Department of Ophthalmology, 75 Arch Street, Akron, USA

This issue is the final publication for the Middle East and African Journal of Ophthalmology for 2010. The year has been a significant one for the journal being listed in PubMed and receiving a record number of submissions. The current edition accurately reflects the scope of the journal that includes publishing studies focusing on ophthalmic disease and challenges in the Middle East and African (MEA) region. In addition, the mission of the journal is to provide up-to-date reviews on recent treatment trends in ophthalmology. The two review publications describe the use of biologicals in the treatment of intraocular inflammation^1^ and an overview on ab interno trabeculotomy in the treatment of glaucoma.^2^ Recently, a wide array of procedures have been developed as an alternate to trabeculectomy for the treatment of glaucoma. These procedures appear to provide reasonable intraocular pressure control with fewer long-term complications. It would be important to study the comparative effectiveness of procedures such as the Trabectome™ in the region where follow-up following glaucoma surgery remains a challenge.

A number of manuscripts in this issue from Africa and the Middle East focus on a wide array of visual impairment related topics. Of particular interest are the issues surrounding childhood visual impairment in the region. The paper by Vora and colleagues^3^ describe higher prevalence uncorrected refractive errors in special-needs children in Oman and Mohammed and colleagues^4^ describe the causes of blindness in a district in north western Nigeria. Furthermore, Ademola-Popoola and co-authors^5^ in an interesting study describe the psychosocial aspects of blindness in a Nigerian city. The common thread that connects these excellent papers under a common theme is the issue of childhood blindness in the MEA region. Pediatric blindness accounts for a significant proportion of blindness in developing countries. In the year 2000, it was estimated that 1.4 million children were blind worldwide and of that figure 50% of blindness was preventable.^6^ In the year 2010, these manuscripts in MEAJO suggest that this significant public health problem still remains unresolved. Agarwal and colleagues have recently reported that the availability of tertiary eye care health facilities for children in Africa was variable and probably inadequate and they suggested specific measures to address this problem.^7^

Two reports focus on serious eye health hazards caused by the use of traditional medications in the region. Blinding eye disease caused by a variety of indigenous herbs and chemicals has been reported for over many years from the Middle East, Africa and Asia. Though the use of alternative medicine has exploded worldwide and probably in some areas of treatment has demonstrated excellent benefit, it must be recognized that the benefit of such traditional medications as therapeutic agents must be determined after evidence-based studies demonstrating effectiveness and safety.^8^ The abuse of blinding traditional medications is still a significant challenge in the region and can be solved only by unique tools that are developed in the region to educate the public on the risks of using such therapy, access to eye care and affordable medications.

Finally, Alwadani and co-authors^9^ address the issue of ophthalmic practice trends in Saudi Arabia. Studies such as these are important in determining events that influence the decision of ophthalmologists in training and their career choices. Careful studies determining the ophthalmology workforce in various regions of the Middle East and Africa are critically important for planning for future needs at the national and regional level. I hope that the articles in this issue will stimulate researchers in the region to collaboratively work toward providing solutions to these challenges within the region.

## References

[1] Neri P, Lettieri M, Fortuna C, Manoni M, Celani S, Giovannini A (2010). Adalimumab (Humira™) in Ophthalmology: A Review of the Literature. Middle East Afr J Ophthalmol.

[2] Pantcheva MB, Kahook MY (2010). Ab Interno Trabecolutomy. Middle East Afr J Ophthalmol.

[3] Vora U, Khandekar R, Natrajan S, Al-Hadrami K (2010). Refractive error and visual functions in children with special needs compared with the first grade school students in oman. Middle East Afr J Ophthalmol.

[4] Muhammad N, Maishanu NM, Jabo AM, Rabiu MM (2010). Tracing children with blindness and visual impairment using the key informant survey in a district of North-Western Nigeria. Middle East Afr J Ophthalmol.

[5] Ademola-Popoola DS, Tunde-Ayinmode MF, Akande TM (2010). Psychosocial characteristics of totally blind people in a Nigerian city. Middle East Afr J Ophthalmol.

[6] Maida JM, Mathers K, Alley CL (2008). Pediatric ophthalmology in the developing world. Curr Opin Ophthalmol.

[7] Agarwal PK, Bowman R, Courtright P (2010). Child eye health tertiary facilities in Africa. J AAPOS.

[8] Tapsell LC, Hemphill I, Cobiac L, Patch CS, Sullivan DR, Fenech M (2006). Health benefits of herbs and spices: The past, the present, the future. Med J Aust.

[9] Alwadani F, Alrushood A, Altokhy H, Alasbali T (2010). A forecast of ophthalmology practice trends in Saudi Arabia: A survey of junior residents. Middle East Afr J Ophthalmol.

